# Risk of Asthmatic Episodes in Children Exposed to Sulfur Dioxide Stack Emissions from a Refinery Point Source in Montreal, Canada

**DOI:** 10.1289/ehp.0800010

**Published:** 2008-10-21

**Authors:** Audrey Smargiassi, Tom Kosatsky, John Hicks, Céline Plante, Ben Armstrong, Paul J. Villeneuve, Sophie Goudreau

**Affiliations:** 1 Institut National de Santé Publique du Québec, Département de santé environnementale et santé au travail, Montréal, Québec, Canada;; 2 Centre de recherche Léa Roback, Université de Montréal, Montréal, Québec, Canada;; 3 Direction de Santé Publique de Montréal, Montréal, Québec, Canada;; 4 School of Occupational and Public Health, Ryerson University, Toronto, Ontario, Canada;; 5 Public and Environmental Health Research Unit, London School of Hygiene and Tropical Medicine, London, United Kingdom;; 6 Biostatistics and Epidemiology Division, Health Canada, Ottawa, Ontario, Canada

**Keywords:** asthma, case crossover, children, dispersion modeling, emergency department visits, hospital admissions, point source, refinery, short-term exposure, sulfur dioxide

## Abstract

**Background:**

Little is known about the respiratory effects of short-term exposures to petroleum refinery emissions in young children. This study is an extension of an ecologic study that found an increased rate of hospitalizations for respiratory conditions among children living near petroleum refineries in Montreal (Canada).

**Methods:**

We used a time-stratified case–crossover design to assess the risk of asthma episodes in relation to short-term variations in sulfur dioxide levels among children 2–4 years of age living within 0.5–7.5 km of the refinery stacks. Health data used to measure asthma episodes included emergency department (ED) visits and hospital admissions from 1996 to 2004. We estimated daily levels of SO_2_ at the residence of children using *a*) two fixed-site SO_2_ monitors located near the refineries and *b*) the AERMOD (American Meteorological Society/Environmental Protection Agency Regulatory Model) atmospheric dispersion model. We used conditional logistic regression to estimate odds ratios associated with an increase in the interquartile range of daily SO_2_ mean and peak exposures (31.2 ppb for AERMOD peaks). We adjusted for temperature, relative humidity, and regional/urban background air pollutant levels.

**Results:**

The risks of asthma ED visits and hospitalizations were more pronounced for same-day (lag 0) SO_2_ peak levels than for mean levels on the same day, or for other lags: the adjusted odds ratios estimated for same-day SO_2_ peak levels from AERMOD were 1.10 [95% confidence interval (CI), 1.00–1.22] and 1.42 (95% CI, 1.10–1.82), over the interquartile range, for ED visits and hospital admissions, respectively.

**Conclusions:**

Short-term episodes of increased SO_2_ exposures from refinery stack emissions were associated with a higher number of asthma episodes in nearby children.

Little is known about the risks associated with exposure to petroleum refinery emissions in young children or, indeed, across other age ranges. Furthermore, studies performed near point sources such as refineries have generally not addressed the effects of short-term exposures (e.g., Bhopal et al. 1998; Forastiere et al. 1994; Kim et al. 2001).

Refinery emissions may be an important source of sulfur dioxide on a local scale (Gower et al. 2008). The potential for short-term high-level SO_2_ exposures to cause adverse health effects is well recognized. High SO_2_ levels were implicated in the acute morbidity and mortality associated with the severe pollution episodes in Donora (Pennsylvania), London, and New York in the 1940s, 1950s, and 1960s (American Thoracic Society Committee of the Environmental and Occupational Health Assembly 1996). Moreover, experimental SO_2_ chamber studies have shown that adult asthmatics experience pronounced airway resistance after exercise after only minutes of exposure at levels similar to those encountered today in ambient air (Carlisle and Sharp 2001).

Epidemiologic studies of children have not demonstrated convincing evidence that daily increases in ambient SO_2_ exposure at typical current levels are associated with respiratory effects (e.g., Ko et al. 2007; Lee et al. 2002). Time-series analyses based on administrative databases have shown only modest increased risks of emergency department (ED) visits and hospitalizations for respiratory problems; usually the increase was < 10% per increase in the inter-quartile range of SO_2_ (e.g., Hajat et al. 1999; Lee et al. 2002; Luginaah et al. 2005; Sunyer et al. 1997; Wong et al. 2001). Other time-series and case–crossover analyses have found no association between ambient SO_2_ levels and ED visits or hospital admissions, or that the risk apparently associated with ambient SO_2_ disappeared after adjusting for the effect of other pollutants that were correlates of SO_2_ (e.g., Fusco et al. 2001; Lee et al. 2006; Lin et al. 2005; Sunyer et al. 2003). Only modest short-term effects of SO_2_ exposure were noted in panel studies of children (e.g., Aekplakorn et al. 2003; Henry et al. 1991; Schildcrout et al. 2006).

To date, epidemiologic studies that have investigated the acute risks of daily ambient SO_2_ in children have been performed mostly in urban areas, with the aim of capturing associations with variable levels of regional air pollution (e.g., Hajat et al. 1999; Lee et al. 2006; Sunyer et al. 1997). Few studies have been performed near SO_2_ point sources (e.g., Aekplakorn et al. 2003; Henry et al. 1991). Because SO_2_ levels are higher near sources and vary widely in space, few studies could have estimated SO_2_ levels at a spatial level fine enough to represent the variability of individual exposure. Indeed, most of the above studies have simply used a small number of centrally located SO_2_ monitors to attribute a measure for an entire region, rather than attempt to capture intraregional variation.

Unlike past work, this study uses dispersion modeling to assign ambient pollution levels at an individual level. Incorporating information on both the spatial (geographic) and temporal variability of SO_2_ levels likely improves characterization of individual exposures for community epidemiologic studies. Dispersion modeling approaches can be used to assign individual exposures that vary in time and space. Dispersion models are physical deterministic models that use existing data characterizing source emissions, meteorology, and topography to create maps of pollutant concentrations. Such maps can be used to predict source-specific exposures outside residences and to provide spatially resolved exposure for examining source-specific health effects. They are usually based on Gaussian plume dispersion equations [U.S. Environmental Protection Agency (EPA) 2008]. Although dispersion models overcome the limitation of central-site measurements that do not allow for the consideration of the spatial variation of pollutant levels, they have seldom been used in epidemiologic investigations and, as far as we know, have not previously been used to assess effects associated with short-term variations in exposure.

The present study is part of a larger assessment of possible health effects from time-variant exposure to emissions from the industrial complex of the East End of Montreal (Canada) where two petroleum refineries are located. In residential areas located near this industrial complex, annualized rates of hospitalizations for respiratory health conditions among children 2–4 years of age were approximately 25% higher than rates for Montreal Island children for 1996–2004 (Kosatsky et al. 2004). However, such a cross-sectional assessment could not establish if the excess is related to exposure to emissions from the industrial complex and, if so, whether the increased rates are related to short- or long-term exposures.

In this study, we used a case–crossover design to assess the risk of asthma episodes in children 2–4 years of age exposed to SO_2_ refinery stack emissions from the industrial complex located in the East End of Montreal Island. According to the Canadian pollutant release inventory (Environment Canada 2008) and the emission inventory of the city of Montreal (Kosatsky et al. 2004), during the study period the two refineries emitted at least 8,000 metric tons of SO_2_ annually. Peak daily SO_2_ levels in residential areas near to the industrial complex can reach values that are > 50% higher than daily peaks measured in other areas of the city and in other Canadian cities such as Toronto, whereas mean levels are in the range typically measured in North American cities and are much lower than in developing countries concentrations (World Health Organization 2000). The specific objectives of this study were *a*) to assess in young children the risk of asthmatic episodes related to short-term exposure to refinery stack emissions as indicated by SO_2_, *b*) to explore the influence of the SO_2_ “exposure regime” using daily means and peaks at various lags, and *c*) to explore the value of a dispersion model versus fixed-site SO_2_ monitors in characterizing exposure to a point source.

## Materials and Methods

### Study Area, Period, and Population

**Figure 1:**
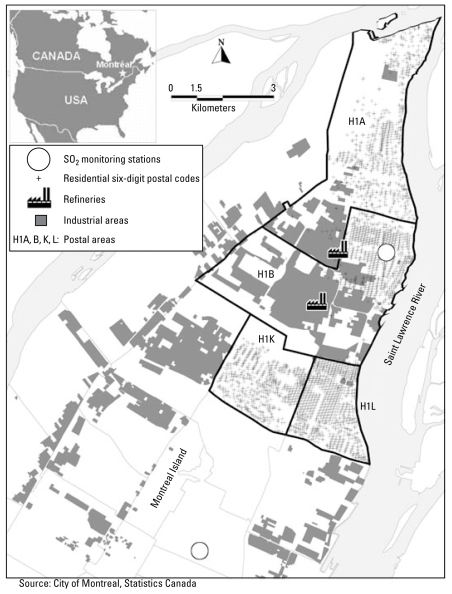
Area of the study, location of refineries and industrial area, SO_2_ fixed-site monitors, three-character postal areas, and residential six-character postal codes. A six-character postal code represents a segment of road (block side) within which fewer than 50 individuals live.

This study covers the calendar period between 1996 and 2004. We defined the geographic area of the study by four postal service forward sortation areas (FSAs) of the East End of Montreal Island (H1A, H1B, H1K, H1L), Canada, where both residences and an industrial sector comprising two petroleum refinery complexes are located. Figure 1 presents the location of the four FSAs: H1A, H1B, H1K, and H1L. We estimated the geographic location of all residences within the four FSAs using the geographic centroids of their full six-character postal codes. Residences within these four FSAs are located as close as 0.5 km and up to 7.5 km from the refinery stacks. The median distance from the 3,469 six-character postal code centroids of all residences to the closest refinery stack is 2.4 km. The Island of Montreal has more than 30,000 residential postal codes; within the study area, a six-character postal code often corresponds to a single segment of road within which fewer than 50 individuals live. Figure 1 shows the location of the centroids of the six-character postal codes in the study area.

The study population consisted of children who lived in one of the four FSAs who either visited an ED or were hospitalized for asthma between 1996 and 2004. We restricted analyses to children who were between 2 and 4 years of age at the time of their ED visit or hospitalization. Estimates of the number of children in this age range who resided in each of the four FSAs, based on the 2001 Canadian Census, were 960, 635, 1,020, and, 855 for FSAs H1A, H1B, H1K, and H1L, respectively. This project obtained approval from the Montreal Public Health Research Ethics Board.

### Health Data

We obtained hospital ED visits and hospital admissions data for children living on the Island of Montreal, Canada, from the Quebec Health Insurance Board and the Quebec Ministry of Health and Social Services (MED-ECHO database), respectively, for the period 1996–2004. These two databases capture virtually all hospitalizations and ED visits for Quebec residents (Labrèche et al. 2008). Each ED visit or hospitalization included the following individual-level information: time and date of the health service used, the primary cause of the ED visit or hospitalization [*International Classification of Diseases, 9th Revision* (ICD-9; World Health Organization 1975)], and patient characteristics such as age, sex, and the six-character postal code of the current place of residence. The location of the hospital where the service took place was available for admission but not for ED visit data. These data indicated that 96% of hospital admissions among children who lived in the four FSAs were at institutions on the Island of Montreal or at its near eastern suburb; we expect a similar figure for ED visits.

We used asthma (ICD-9 code 493) as the cause of ED visit or hospitalization for the analyses. We included children ≥ 2 years of age because the diagnosis of asthma among younger children is problematic and often confused with bronchiolitis, viral infections, or other conditions that produce wheeze (Panettieri et al. 2008). This study focused on asthma outcomes among very young children because cross-sectional analyses revealed a more pronounced excess of asthma hospitalizations for this age group than for others, and because children 2–4 years of age are widely regarded to be more susceptible to possible adverse health effects of air pollution (Bateson and Schwartz 2008).

### Pollution and Meteorological Data

Our primary exposure of interest was the SO_2_ emitted by the refineries and estimated at the child’s residence. In our assessment of the effects of emissions from the refineries of the East End of Montreal, we have considered the acute effects of exposure to SO_2_ because *a*) SO_2_ has been associated with adverse acute health outcomes in other studies and *b*) it serves as an indicator of stack emissions from the refineries. Of course, stack-emitted SO_2_ is likely to be correlated with other refinery emissions such as fine particles [particles with an aerodynamic diameter < 2.5 μm (PM_2.5_)].

We used two fixed monitoring sites located near the refineries and estimates from the AERMOD (American Meteorological Society/Environmental Protection Agency Regulatory Model) atmospheric dispersion model (U.S. EPA 2008) to characterize ambient SO_2_ exposures of children who visited the ED or who were admitted to hospital between 1996 and 2004. We also characterized exposure of the children to the “urban/regional background” levels of air pollutants with the use of all Montreal Island’s fixed monitoring sites with available air pollutant data, excluding the two sites located near the refineries (see “Regional/urban air pollutant background levels,” below). We computed daily SO_2_ means and maxima from hourly measurements at fixed-site monitors and from hourly model estimates at 3,469 receptor locations (from 0000 to 2300 hours). We used daily maxima to allow us to represent the possible respiratory health effects from high-exposure episodes that occur during the day. We considered days with < 18 hr of SO_2_ measurement as missing.

#### Fixed-site measurements of SO_2_ levels near refineries

We considered daily SO_2_ measurements made at the following locations by the Montreal Environmental Service and that capture the refinery emissions (see Figure 1 for location). First, to represent SO_2_ exposure for those living in the FSAs HIA and H1B, we used data from the monitor close by and to the east of the refineries. Based on hourly wind data, winds blow from the refineries toward this monitor (winds from 210° to 290°) about 40% of the time. This monitor measured the highest SO_2_ levels for winds from 210° to 290° (average levels with winds from 210° to 290°, 10.9 ± 13.0 ppb). This monitor is located within 3 km of the refinery stacks. The maximum distance from this monitor to the residences (more precisely, to the six-character postal codes) of these FSAs is 6.9 km; the median distance is 1.6 km.

Second, to represent SO_2_ exposure for those living in the FSAs H1K or H1L, we used data from the monitor close by and to the southwest of the refineries. Winds blow from the refineries toward this monitor (winds from 0° to 60°) about 20% of the time (mostly in winter). This monitor is located outside of the four FSAs of the study and no more than 10 km from the refinery stacks. It can be as far as 9.1 km from some residences of the H1K and H1L FSAs (the median distance is 5.6 km). Although this monitoring site is far from many of the residences of these FSAs, SO_2_ levels measured at this monitor are influenced by the refinery stack emissions when the wind is from the northeast (average levels with winds from 0° to 60°, 7.7 ± 10.6 ppb). No other SO_2_ monitoring site was located closer to FSAs H1K and H1L.

**Figure 2:**
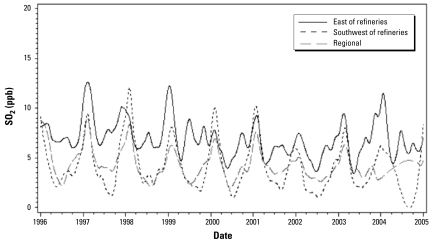
Time series of daily mean SO_2_ measurements at fixed sites on the Island of Montreal (spline routing smoothing). Peaks at the southwest monitoring site occurred during winter.

Figure 2 presents the time series of the SO_2_ levels at these two monitors.

#### Dispersion modeling of SO_2_ levels

To obtain at-home estimates of daily exposure, we estimated SO_2_ levels at the centroid of all children’s residential six-character postal codes for all days between 1996 and 2004, using the AERMOD dispersion model as follows.

We used data for several point source emissions of the two refineries to model hourly SO_2_ levels at 3,469 discrete receptor locations corresponding to the residential six-character postal code centroids in the East End of Montreal (in the FSAs H1A, H1B, H1K, H1L; Figure 1). The point source emissions included those from main vents and stacks that emit on a continuous schedule throughout the year (seven point-source emission sources for one refinery and five for the other). For each vent and stack, the longitude/latitude, emission temperature, height, and exit velocity were available. The selected vent and stack emissions of the two refineries represent approximately 90% of the total SO_2_ emissions from these two refineries and represent more than 80% of the SO_2_ emissions in the industrial area. We used annual emission data for each vent and stack to model hourly SO_2_ levels. Although information on daily variation of SO_2_ emissions was not available, monthly variation of total SO_2_ refinery emissions was available for each facility for the study period (all point sources for each refinery considered together). We thus adjusted annual emissions data for the month-by-month emission levels of each facility, using the monthly variation of their total SO_2_ refinery emissions.

Other smaller point sources of SO_2_ are on Montreal Island, and additional “regional sources” are outside of Montreal. We did not represent these in the model, but we explored their influence on the risk of ED visit or hospitalization for asthma in children using regression models that incorporated the SO_2_ regional/urban background levels.

The inputs to the dispersion model also included hourly meteorological records at the Pierre Elliott Trudeau Montreal International Airport, about 25 km from the area of the study, and upper air data from a rural monitoring site descriptive of the greater Montreal region. Missing hourly meteorological data at the International Airport monitoring site (< 0.1% of all hours) were replaced with the values for the previous or the next hour or interpolated from available data (when more than a few consecutive hours were missing). We acquired all meteorological data from Environment Canada (2008). The topographic characteristics across the area of interest were considered constant. We made allowances in the model for the nature of the local terrain, including both vegetated (grass) and paved surfaces.

We averaged hourly SO_2_ predictions over the day and computed daily peaks for each six-character postal code. We linked daily SO_2_ values with the case and control period intervals by date and by postal code.

#### Regional/urban air pollutant background levels and regional meteorological data

##### Pollutant measurements

The fixed-site monitors used to create hourly regional/urban background averages were not located in the immediate vicinity of the industrial complex. Of these fixed-site monitors, we used at least six for nitrogen dioxide and ozone, depending on the date; we used three monitors for SO_2_ measurements and only one for PM_2.5_. We used the three SO_2_ monitors not located near the industrial sector to represent minor point sources of SO_2_ on Montreal Island and additional regional sources outside of Montreal. The three monitors were influenced by the local refinery emissions when winds were from the northeast (0–60°) but to a lesser extent than the monitor also located to the west but in close proximity to the refineries. Because levels measured at the three monitors were not influenced by other wind directions, we presume that although these monitors may also capture emissions from other local sources, such influence is small compared with that from outside the Island of Montreal.

#### Meteorological measurements

We used daily mean outdoor temperatures and relative humidity (from 0000 to 2300 hours) from the Montreal International Airport.

## Data Analysis

We analyzed the study using a case–crossover design (Maclure 1991). In this design, which is analogous to a standard time-series design, we controlled for secular trends in hospital morbidity by selecting control days for each day in which one or more hospital visits (case day) occurred. This design also controls for time-invariant confounders (e.g., exposure to secondhand smoke) by making within-subject comparisons. We selected control days using the time-stratified approach (Lumley and Levy 2000) in which we divided the study period into monthly strata and selected control days for each case as the same days of the week in the month. The time-stratified approach removes bias from unwanted secular trends in the hospitalization/ED visit time series and leads to unbiased estimates of effect for case–control days selected within specific time windows (no “overlap bias”) (Janes et al. 2005). Thus, if hospitalization occurred on Saturday, 22 August 1998 (corresponding to the hazard period, lag 0), the selected control periods were also Saturdays (1, 8, 15, and 29 August 1998).

We assessed the SO_2_–hospitalization/ED visit relationships by conditional logistic regression analyses in which we compared the SO_2_ exposure levels (AERMOD estimates or the fixed-site measurements near the refineries) for the case period with the matched control periods. We defined the case period as the day of hospitalization or ED visit. Exposure metrics examined included same-day average and peak SO_2_ exposures, as well as lagged intervals extending from 1 to 4 days before the case or control event. We estimated the odds ratios (ORs) and their 95% confidence intervals (CIs) in relation to an increase in the interquartile range of SO_2_.

Analyses performed with the SO_2_ levels from the monitoring sites were done separately for children residing in the East End (including the FSAs H1A and H1B) and to the southwest (including H1K and H1L) of the refineries, as the two SO_2_ fixed monitoring sites used, to represent the exposures of children living to the east or the southwest of the refineries, which differ the emission sources and to the residences of the children (Figure 1). As such, the SO_2_ levels measured at the site farther away from the refineries are typically lower than those at the closer monitoring site (Figure 2). Thus, the two SO_2_ fixed monitoring sites may not represent the exposure of children living to the east or the southwest of the refineries with equal accuracy. When using estimates from AERMOD, we performed analyses for the two areas separately, to compare results with those from analyses performed with the SO_2_ levels from the two monitoring sites. However, we also performed analyses with AERMOD SO_2_ estimates for all FSAs together, to increase the statistical power of the overall SO_2_ effect estimate.

We first performed unadjusted conditional logistic models by including the AERMOD SO_2_ exposure estimates or the fixed-site measurements near the refineries as an independent variable. We then used multivariable conditional logistic regression models to control for the potential confounding influences of the regional/urban background air pollutant levels and meteorological conditions. We adjusted for daily mean concentrations of regional SO_2_, O_3_, NO_2_, and PM_2.5_ levels, evaluated for the same lag period as for the SO_2_ AERMOD estimates and the fixed-site measurements near the refineries. Similar to previously conducted time-series and case–crossover analyses (e.g., Luginaah et al. 2005), meteorological conditions that we examined included relative humidity and temperature at the same lag period as SO_2_ exposure estimates. We verified the linearity of the relationship between temperature and ED visits or hospitalization using natural cubic spline functions. We constrained cubic basis functions to meet at the following cut points (knots): 5th, 33th, 66th, and 95th percentiles. Given no departure from linearity, we used linear relations.

We compared dispersion model predictions (SO_2_ AERMOD daily mean and peak estimates) with measurements at the SO_2_ fixed-site monitor located to the east of the refineries using Pearson correlations.

## Results

During the study period, there were 263 hospitalizations and 1,579 ED visits for asthma among children 2–4 years of age in the four FSAs of the study; 46% and 41% of the hospitalizations and of the ED visits were among children who lived to the east of the refineries (FSAs H1A and H1B). Based on 2001 census data (Statistics Canada 2008), these two FSAs accounted for a combined 46% of the overall population of the study area. The daily number of hospitalizations and ED visits ranged from 0 to 3 (mean, 0.1 ± 0.3) and from 0 to 5 (mean, 0.5 ± 0.7), respectively, during the study period.

Table 1 presents SO_2_ descriptive data for the fixed-monitoring sites located near and to the east and southwest of the refineries, the AERMOD SO_2_ estimates for all the receptor points located to the east or to the southwest of the refineries for all days of the study period, and the SO_2_ AERMOD descriptive information for the four FSAs combined. Descriptive SO_2_ data including only dates for those who used a health service were similar (data not shown) to results presented in Table 1. The monitor to the east of the refineries presented higher SO_2_ levels than those measured at the monitor to the southwest, which is both farther away from the refineries and less frequently in the lee of refinery stack emissions. When both monitors were not subjected to winds blowing over the refineries, levels recorded were similar to those of the urban/regional background (data not shown). The distribution of the AERMOD estimates presents a lower mean than do the monitors. However, peak levels measured to the west of the refineries are lower than the AERMOD peak level estimates. As for the urban/regional background levels, SO_2_ measurements showed limited variability compared with the levels recorded at the monitoring sites located near the refineries. NO_2_ and O_3_ levels monitored in proximity and to the east or southwest of the refineries were not higher than the urban/regional background levels (data not shown).

Correlation of the dispersion model predictions (SO_2_ AERMOD daily mean and peak estimates) at the site of the fixed monitor located to the east of the refineries with SO_2_ measured at that monitor was modest (daily mean, *r* = 0.43; peak, *r* = 0.36; *p* < 0.001).

Table 2 presents associations between AERMOD SO_2_ estimates and hospitalizations and ED visits for children from analyses performed using combined data from the east and the southwest areas. We performed such combined analyses only for the SO_2_ AERMOD estimates, given that the two fixed monitoring sites used to represent exposure in the east and in the southwest are located at different distances from the refineries and residences of the FSAs. Combined data show a greater OR for hospitalizations than for ED visits. Hospitalizations and ED visits were more strongly related to peak than to mean levels of SO_2_; associations were most pronounced for SO_2_ levels on the same day that the visit or the hospitalization took place (lag 0).

Adjusting for daily ambient temperature, relative humidity, and the daily regional/urban background of SO_2_, O_3_, and NO_2_ had little influence on the associations observed between SO_2_ levels from refinery stack emissions and ED visits and hospitalizations (Table 2). Adjustment for the regional/urban background of SO_2_ “overcontrolled” for industrial SO_2_ stack emissions, given its correlation with AERMOD SO_2_ levels: The “background” of SO_2_ was the ambient air pollutant most correlated to AERMOD estimates, but even this correlation was only moderate (AERMOD daily mean SO_2_ estimates of all postal codes and daily mean background SO_2_ levels, *r* = 0.30). Adjustment for the daily regional/urban background of PM_2.5_ also had little influence on the associations observed. We did not adjust the ORs presented in Table 2 for PM_2.5_ levels because measurements were missing for 1996 and 1997.

**Figure 3:**
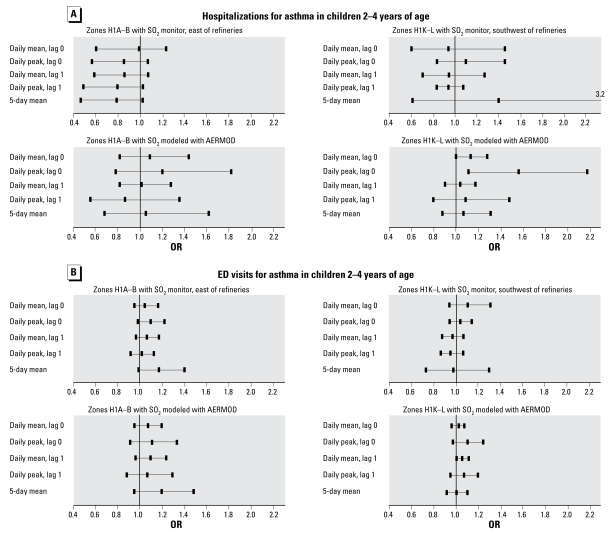
Adjusted associations between SO_2_ measurements at fixed-site monitors (located to the east and southwest of refineries) or SO_2_ concentrations estimated with AERMOD, and hospitalizations (*A*) and ED visits (*B*) for asthma in children 2–4 years of age living near (to the southwest or east) of the refineries on the Montreal Island, between 1996 and 2004. We adjusted for daily ambient temperature, relative humidity, and daily regional/urban background of SO_2_, O_3_, and NO_2_. ORs are expressed as increments of the interquartile range (Table 1). The SO_2_ measurement site used to assess health risks in the postal areas H1K and H1L is farther away from refineries than is the monitoring site used for H1A and H1B (see Figure 1).

Figure 3 presents the adjusted associations between the SO_2_ levels, measured or estimated with AERMOD, for the FSAs located to the east (H1A and H1B) and to the southwest (H1K and H1L) of the refineries, for the different exposure variables and lags, and hospitalizations and ED visits for asthma in children. The associations between SO_2_ levels from fixed monitoring sites and hospitalizations for asthma in children do not show consistent evidence for adverse effects. Analyses with SO_2_ levels modeled with AERMOD show that peak levels at lag 0 are associated with hospitalizations for asthma in children to the east but mostly to the southwest of refineries. Inconsistent results obtained with SO_2_ levels measured or estimated with AERMOD may be attributable to the small number of hospitalizations of children in these four FSAs (*n* = 122 east and *n* = 141 southwest of refineries). For ED visits, ORs derived using SO_2_ levels from the fixed monitoring sites or estimated with AERMOD followed similar tendencies only in the FSAs located to the east of the refineries (H1A and H1B): There were elevated ORs which were somewhat more pronounced for daily peak at lag 0 and for the 5-day mean SO_2_ levels (Figure 3). For example, the OR for visiting an ED for asthma, for the interquartile range in the peak SO_2_ levels (23 ppb) measured at the fixed site at lag 0, is 1.09 (95% CI, 0.98–1.21) for children living to the east of the refineries (Figure 3B, top left). For these children, the OR for visiting an ED for asthma for an interquartile range in the peak SO_2_ levels estimated with AERMOD at lag 0 (32 ppb) is 1.10 (95% CI, 0.91–1.33; Figure 3B, bottom left).

## Discussion

In this study we assessed the risk of asthma episodes in children exposed at their residence to SO_2_ stack emissions from a refinery point source, using a case–crossover design. This is the first use of this design to assess the risks associated with point source emissions. The use of such a design allowed us to address the effects of short-term exposures. The study shows a modest association of same-day SO_2_ levels with asthma ED visits and hospitalizations among children living near refineries. Risks appear more pronounced for daily SO_2_ peak levels than for mean levels.

Little information is available to compare the above results on risks of childhood asthma episodes associated with SO_2_ from a point source, because most studies of SO_2_ and asthma have been performed in urban areas with the aim of establishing relationships between daily SO_2_ levels and asthma episodes (e.g., Hajat et al. 1999; Lee et al. 2006; Lin et al. 2005; Schildcrout et al. 2006; Sunyer et al. 1997; Wong et al. 2001). Nonetheless, short lags have been demonstrated in a number of studies on asthma episodes related to other air pollutants such as PM_2.5_ (U.S. EPA 2004). The results of our epidemiologic assessment are also concordant with those of experimental studies where exercising humans were exposed on a short-term basis to high levels of SO_2_. Such studies have shown that some asthmatics experience change in pulmonary functions and respiratory symptoms after a peak exposure as short as 10 min (Carlisle and Sharp 2001).

Studies that noted a positive association between ED visits or hospitalizations for asthma and acute exposure to SO_2_ have usually reported risks < 10% per SO_2_ inter-quartile range of magnitude similar to or greater than in our study (e.g., Hajat et al. 1999; Lee et al. 2002; Luginaah et al. 2005; Sunyer et al. 1997; Wong et al. 2001). Such risks are similar to what we observed in the present study for ED visits; however, relative risks for hospitalizations appeared greater in the present study.

In this study, we estimated exposure to refinery stack emissions using both SO_2_ fixed-site measurements and the AERMOD atmospheric dispersion model. SO_2_ fixed-site monitors may not adequately represent exposure to refinery stacks emissions if located too far from the emission source. In our study, associations between ED visits and SO_2_ from the fixed monitor located to the east of the refineries provided results similar to those with AERMOD estimates. On the other hand, associations between ED visits and exposures represented by the southwest monitor, which is more distant from the residences of interest, diverged with associations with the AERMOD estimates. Dispersion models may be useful to represent point source exposures in areas where there is no local monitor or where monitors are not close enough to appropriately represent exposure to emission sources. However, modeling errors associated with the source information (e.g., monthly emission data to compute daily estimates, use of upper air data from a location hundreds of kilometers away from the study area) and the limitations of the model to represent specific dispersion conditions should not be disregarded. As noted in Table 1, AERMOD SO_2_ estimates present lower mean levels than those measured at fixed monitoring sites, because we omitted from the model SO_2_ emission sources other than refinery emissions.

Several other limitations are associated with the estimates of SO_2_ used in this study. First, SO_2_ levels estimated at the residence of children may not adequately represent exposure because children are not always at home. However, children 2–4 years of age are more likely to be present in their neighborhood than are older children who attend school. Second, our SO_2_ exposure estimates, which represent expected ambient residential SO_2_ levels, may not adequately represent exposure because children are likely to spend most of their time indoors. SO_2_ levels are lower indoors because absorption occurs on walls, furniture, and ventilation systems (World Health Organization 2000).

Finally, the associations observed in the present study, if causal, probably represent the effects of short-term exposures to a pollutant mixture, even after statistical control for the urban/regional background of pollutant levels. Indeed, the SO_2_ exposure estimates used in this study are likely correlated with other stack and/or fugitive refinery emissions such as PM_2.5_ and volatile organic compounds. From a policy perspective, we need to better disentangle their effects to determine which emissions to control.

## Conclusion

We initiated this study to clarify whether higher hospital admission rates for respiratory problems for children in the East End of Montreal were related to short-term variations in refinery emissions. Our results suggest that same-day SO_2_ peak levels, rather than daily mean levels, were associated with asthmatic episodes in young children who lived in close proximity to the refineries.

## Figures and Tables

**Table 1:** SO_2_ near refineries, regional air pollutants, and meteorological data for Montreal Island, 1996–2004.

Pollution variable	No. of days	Mean	SD	Median	Q1	Q3	IQR
SO_2_ measured (ppb)^a^							
Monitoring site east of refineries							
Daily mean	3,177	6.9	5.7	5.5	2.9	9.2	6.3
Daily peak	3,177	23.8	22.2	16.9	8.9	31.9	23.1
5-day mean (lag 0 to lag 4)	3,055	6.9	3.5	6.3	4.4	8.7	4.4
Monitoring site southwest of refineries							
Daily mean	2,683	4.4	4.4	3.1	1.5	5.9	4.3
Daily peak	2,683	12.8	13.8	8.5	4.2	16.2	11.9
5-day mean (lag 0 to lag 4)	2,561	4.5	3.2	3.7	2.1	5.9	3.7
SO_2_ modeled with AERMOD (ppb)							
East and southwest of refineries^b^							
Daily mean	11,406,072	3.0	4.8	0.8	0.0	4.3	4.3
Daily peak	11,406,072	17.5	20.0	10.8	0.0	31.2	31.2
5-day mean (lag 0 to lag 4)	11,392,196	3.0	3.2	2.0	0.7	4.3	3.6
East of refineries^c^							
Daily mean	5,592,888	3.7	5.1	1.6	0.0	5.5	5.5
Daily peak	5,592,888	19.2	19.2	16.0	0.1	31.7	31.6
5-day mean (lag 0 to lag 4)	5,586,084	3.7	3.4	2.8	1.0	5.3	4.3
Southwest of refineries^c^							
Daily mean	5,813,184	2.4	4.5	0.2	0.0	3.0	3.0
Daily peak	5,813,184	16.0	20.6	3.3	0.0	30.4	30.4
5-day mean (lag 0 to lag 4)	5,806,112	2.4	2.8	1.5	0.5	3.2	2.7
Regional data^d^							
PM_2.5_ daily mean (μg/m^3^)^e^	2,439	7.6	7.1	5.6	3.0	9.8	6.8
SO_2_ daily mean (ppb)	2,920	4.3	2.9	3.6	2.4	5.3	2.9
NO_2_ daily mean (ppb)	3,257	20.5	7.4	19.5	15.3	24.4	9.2
O_3_ daily mean (ppb)	3,288	17.8	9.1	16.9	11.3	23.0	11.7
Daily temperature (°C)^f^	3,288	7.4	11.7	7.8	−1.5	17.8	19.3
Daily relative humidity^f^	3,177	70.1	12.8	70.5	61.5	79.4	19.9

Abbreviations: IQR, interquartile range; Q1, first quartile; Q3, third quartile. This table presents values for all days and estimates at all locations, even if no ED visits or hospitalizations occurred at these days and places.

a Missing data were spread over the entire study period.

b Number of receptor points (3,469 six-character residential postal codes) × 3,288 days.

c Number of receptor points.

d Average of levels at urban/regional background monitoring sites, excluding monitoring sites east and southwest of refineries.

e Data missing in 1996 and half of 1997.

f Meteorological records from the Montreal International Airport meteorological monitoring site.

**Table 2:** Associations between AERMOD SO_2_ estimates and asthma episodes in small children living near the Montreal refineries, 1996–2004.^a^

	OR (95% CI)	
SO_2_ modeled with AERMOD (μg/m^3^ )	Unadjusted^b^	Adjusted^b^,^C^
Hospital admissions		
Daily mean, lag 0	1.14 (1.02–1.29)	1.14 (1.00–1.30)
Daily peak, lag 0	1.34 (1.08–1.67)	1.42 (1.10–1.82)
Daily mean, lag 1	0.99 (0.88–1.11)	1.03 (0.91–1.16)
Daily peak, lag 1	0.95 (0.75–1.19)	1.01 (0.79–1.29)
5-day mean	1.08 (0.92–1.27)	1.07 (0.87–1.31)
ED visits		
Daily mean, lag 0	1.06 (1.01–1.11)	1.04 (0.98–1.10)
Daily peak, lag 0	1.14 (1.04–1.25)	1.10 (1.00–1.22)
Daily mean, lag 1	1.04 (0.99–1.10)	1.05 (1.00–1.12)
Daily peak, lag 1	1.03 (0.94–1.13)	1.05 (0.95–1.16)
5-day mean	1.05 (0.97–1.14)	1.04 (0.94–1.14)

a Includes children 2–4 years of age living to the east and to the southwest of the refineries.

b ORs are expressed as increments of the interquartile range (see Table 1).

c Adjusted for daily air pollutant regional/urban background levels, regional temperature, and relative humidity, evaluated at the same lag period as SO_2_ estimates from AERMOD.
